# Poisson’s Ratio of Glasses, Ceramics, and Crystals

**DOI:** 10.3390/ma17020300

**Published:** 2024-01-07

**Authors:** Seiji Kojima

**Affiliations:** Division of Materials Science, University of Tsukuba, Tsukuba, Ibaraki 305-8573, Japan; kojima@ims.tsukuba.ac.jp; Tel.: +81-29-874-7728

**Keywords:** Poisson’s ratio, glass, ceramic, crystal, pulse-echo method, ultrasonic resonance, Brillouin scattering, atomic force microscopy, first-principles calculation

## Abstract

Poisson’s ratio is the fundamental metric used to discuss the performance of any material when strained elastically. However, the methods of the determination of Poisson’s ratio are not yet discussed well. The first purpose of this paper is to introduce the five kinds of typical experimental methods to measure Poisson’s ratio of glasses, ceramics, and crystals. The second purpose is to discuss the experimental results on the variation of Poisson’s ratio by composition, temperature, and pressure reviewed for various glasses, ceramics, and crystals, which are not yet reviewed. For example, in oxide glasses, the number of bridging oxygen atoms per glass-forming cation provides a straightforward estimation of network crosslinking using Poisson’s ratio. In the structural-phase transition of crystals, Poisson’s ratio shows remarkable temperature-dependence in the vicinity of a phase-transition temperature. The mechanism of these variations is discussed from physical and chemical points of view. The first-principles calculation of Poisson’s ratio in the newly hypothesized compounds is also described, and its pressure-induced ductile–brittle transition is discussed.

## 1. Introduction

Poisson’s ratio, *ν*, is defined by

(1) $$\nu =-\frac{x_{t}}{x_{l}}$$

where *x_t_* = (∆*d*/*d*) is the lateral contraction ratio of a sample with a transverse size *d* and *x_l_* = (∆*l*/*l*) is the relative longitudinal extension of a sample with a length *l*. In an isotropic material with three dimensions, such as glasses and ceramics, the number of independent elastic moduli is two and it holds that

(2) $$Y=2G\left(1+\nu \right),\;\;\nu =\frac{Y-2G}{2G},$$

where *Y* and *G* are Young’s and shear moduli, respectively. For three-dimensional isotropic materials, it holds that −1.0 ≤ *ν* ≤ 0.5. For compact and weekly compressive materials, such as liquids, stress primarily results in a shape change, and *ν* is close to 0.5. Poisson’s ratio of soft materials, such as rubber, is close to 0.5, while that of porous materials, such as cork, is near 0. For most polymers, ceramics, and metals, 0.25 ≤ *ν* ≤ 0.35, and for gases, *ν* = 0. Materials with different Poisson ratios show very differently mechanical behaviors [1]. 

For structural glasses, the correlation between Poisson’s ratio and fragility (*m*) was reported [2]. The correlation among Poisson’s ratio, fragility, and atomic packing density (*C*_g_) was discussed, and an overall increase in *ν* with an increase in *m* and *C*_g_ was suggested [3].

Auxetic materials with a negative Poisson’s ratio draw increasing attention [4,5]. The auxetic properties were reported in various materials, such as cubic crystals [6] and polymers [7]. Recent first-principles calculations predicted that the Si_2_XY monolayers are auxetic materials with a negative Poisson’s ratio along both *x* and *y* axes [8].

## 2. Experimental Methods to Measure Poisson’s Ratio

Up to the present, several kinds of experimental methods have been used to determine Poisson’s ratio of various glasses, ceramics, crystals, and metals through the measurements of strain, ultrasonic waves, and thermally excited acoustic phonons. In this chapter, five kinds of typical experimental methods, namely (1) the ultrasonic pulse-echo method, (2) resonant ultrasonic spectroscopy, (3) piezo-resonance method, (4) Brillouin scattering spectroscopy, and (5) atomic force microscopy are introduced.

### 2.1. Ultrasonic Pulse-Echo Method

**Figure 1:**
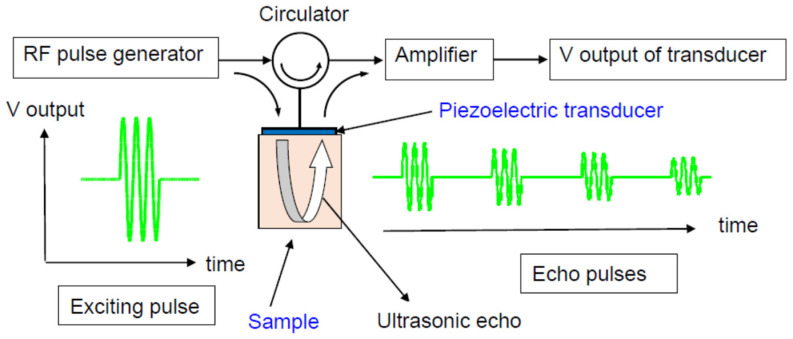
Ultrasonic pulse-echo overlap method [11]. The sound velocity of a sample is determined based on the travel time of an ultrasonic pulse and the travel length of a sample. The operating frequency is usually 1~10 MHz.

The pulse-echo method has been used for nondestructive testing, which is analogous to the sonar system in ships for sounding ocean depths. It is also useful to determine the sound velocity of a sample. The schematic illustration of the ultrasonic pulse-echo method is shown in Figure 1. The ultrasonic/piezoelectric transducer is attached to the top of the sample. The RF pulse is converted to ultrasonic waves into the sample by a piezoelectric transducer and the reflected ultrasonic waves (echo) at the bottom of a sample are converted again to electric output signals by the same piezoelectric transducer. The sound velocity of a sample, *V*, is determined based on the ultrasonic travel time, τ, and the travel length, *L*, using the equation *V* = 2*L*/τ. In the pulse-echo overlap method, the ultrasonic travel time is accurately determined based on the McSkimin criterion to find the condition of correct overlapping between ultrasonic echoes [9,10,11].

### 2.2. Resonant Ultrasonic Spectroscopy

**Figure 2:**
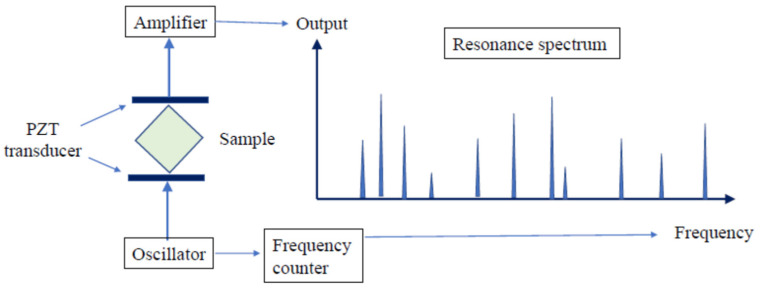
Experimental setup to measure the resonance frequencies of a rectangular parallelepiped sample [18]. Two shear mode transducers are used as an exciting source and detection, where PZT transducers are piezoelectric ceramics. Poisson’s ratio is determined based on the measurement of tortional and spheroidal resonance-mode frequencies.

Resonant ultrasonic spectroscopy (RUS) is a resonance technique that consists of placing a sphere or a cube of a sample on a piezoelectric shear-mode transducer and exciting the vibration of mechanical eigen modes of a sample. From the measured resonance spectrum in the frequency domain and the measured diameter or size, the sound velocity and elastic moduli of a sample are determined. The mode spectrum is assigned based on the comparison of the experimental modes with the calculated values for an ideal isotropic sphere. Eigenvalue modes of an isotropic elastic sphere were calculated analytically by Sato and Usami [12,13,14]. In a sphere, *T*_nm_ denotes the torsional modes, which are pure shear modes; *S*_nm_ denotes the spheroidal modes, which are mixed shear and dilatational modes. The subscripts *n* and *m* indicate the number of oscillation nodes in the radial direction of the sphere and the order of spherical Bessel functions *nm*, respectively. For isotropic materials, elastic moduli, such as Young’s modulus, the shear modulus, and Poisson’s ratio, are determined based on the mode frequencies of *T*_nm_ and *S*_nm_ modes [15]. For a single crystal, all elastic stiffness constants are determined using the cube resonance method [16]. The rectangular parallelepiped resonance was also measured to determine the elastic properties of a small crystal, as shown in Figure 2. In the martensitic transformation of a shape-memory alloy, the elastic anomaly occurs in an austenitic-to-martensite transformation [17]. The free vibration frequencies of a Cu-Al-Ni crystal of a shape-memory alloy are measured as a function of the exciting frequency to determine all elastic stiffness constants [18]. In the superconductor, the elastic constants show the difference between the superconducting and the normal states [19]. The mechanical resonance frequency and amplitude of spherical superconducting Ba(Pb_1−x_Bi_x_)O_3_ ceramics was also measured from 4 K to 300 K with the external magnetic field (0~5 T) to discuss the change in the conduction electron mean path at *T*c = 12 K [20]. It is possible for the conventional RUS to determine all independent elastic constants of a crystal by comparing observed and calculated free-vibration resonance frequencies. However, to avoid invalid local minima in the inverse process, good initial guesses of the elastic constants must be available. Very recently, a deep-learning assisted scheme was proposed to solve this problem, which utilizes an input elasticity image composed of three layers determined from resonance frequency data [21].

### 2.3. Piezoelectric Resonance Method

Poisson’s ratio of piezoelectric materials has been measured using the resonance-antiresonance method. In a piezoelectric material, electric displacement *D* is proportional to a mechanical stress *X*. The sign of *D* changes to the opposite one if the direction of mechanical stress *X* is reversed. Piezoelectric materials also show a converse piezoelectric effect that strain *e* is induced by an electric field *E*. Again, the sign of strain *e* is switched to the opposite one if the direction of an electric field is reversed. Through the application of alternative electric fields to a piezoelectric material, the mechanical vibration is excited by its piezoelectricity [22,23]. 

**Figure 3:**
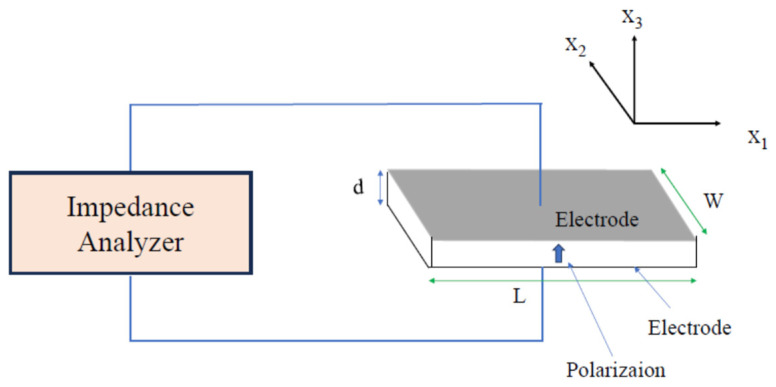
Apparatus for measuring the piezoelectric resonance of a piezoelectric plate with length *L*, width *W*, and thickness *d*.

In the apparatus shown in Figure 3, alternating voltage is applied along the *x*_3_-axis, and polarization is induced along the *x*_3_-axis. Through the piezoelectric effect related to the piezoelectric constant d_13_, the strain is induced along the *x*_1_-axis. The frequency-dependent response from a sample is analyzed by an impedance analyzer. Piezoelectric resonance and antiresonance are observed at the frequencies *f*_r_ and *f*_a_ based on the condition of *Y* = ∞ and 0, respectively, where *Y* is admittance. The resonance frequency is given by

(3) $$f_{r}=\frac{1}{2L\sqrt{\rho s_{11}}},$$

where *L*, *ρ*, and *s*_11_ are the length along the *x*_1_-axis, density, and elastic compliance constant of a sample to be studied, respectively. The electromechanical coupling coefficient *K* is given by.

(4) $$\frac{f_{a}-f_{r}}{f_{r}}=\frac{4}{\pi^{2}}\frac{K^{2}}{1-K^{2}}\;$$

When the electric field, *E*, is along the *x*_3_-axis and the induced strain is along the *x*_1_-axis (lateral mode), it holds in a cubic crystal that.

(5) $$K\equiv \sqrt{\frac{d_{13}}{\varepsilon_{33}s_{11}}},$$

where *d*_13_, *ε*_33_, and *s*_11_ are piezoelectric, dielectric, and elastic compliance constants. The piezoelectric resonance–antiresonance method is an important method to determine the electromechanical coupling constants of piezoelectric ceramics.

In the radial vibration of a disk of piezoelectric ceramics, the electromechanical coupling factor *K_P_* is given by.

(6) $$K_{P}=\sqrt{\frac{2}{1-\nu }}\;\cdot \;\frac{d_{31}}{\sqrt{\varepsilon_{33}^{T}s_{11}^{E}}}$$

Poisson’s ratio is approximately determined as

(7) $$\nu^{E}=\frac{5.332f_{r}-1.867f_{r1}}{0.6054f_{r1}-0.9010f_{r}},\;\mathrm{for}\;0.27<\nu^{E}<0.42,$$

where *f*_*r*1_ is the first overtone frequency [24]. PZT is a well-known piezoelectric material, while it is very difficult to grow single crystals. Therefore, PZT ceramics have been used for the piezoelectric application. The temperature-dependence of Poisson’s ratio of PZT ceramics was reported [25].

### 2.4. Brillouin Scattering Spectroscopy

**Figure 4:**
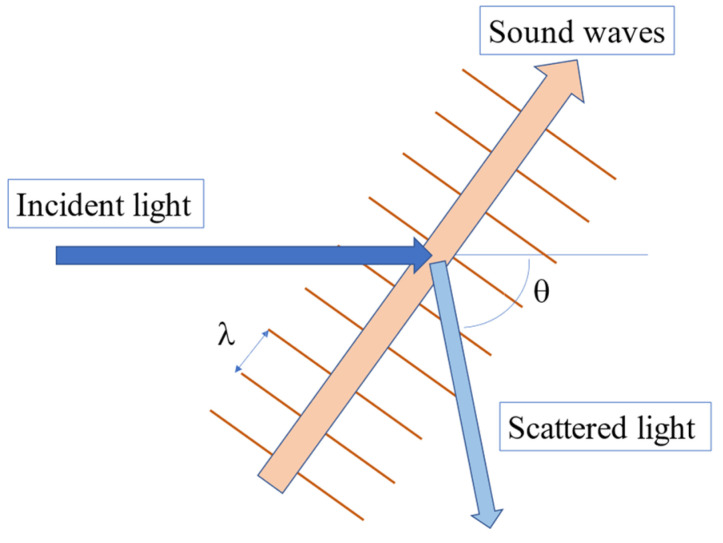
Inelastic light scattering by thermally excited acoustic phonons where λ and *θ* denote the wavelength of the acoustic mode and scattering angle between the incident and scattered light, respectively.

Brillouin scattering spectroscopy is a non-contact and non-destructive method to measure the velocity of acoustic phonons and elastic constants of a material using monochromatic visible light. Brillouin scattering is the inelastic scattering of light by thermally excited acoustic phonons of a sample, as shown in Figure 4. The single-frequency laser beam is incident to a sample to be observed, and the scattered light from a sample is collected into Fabry-Perot interferometers with high-frequency resolution and is detected by a photon-counting system or a CCD detector [26].

The velocity of acoustic phonons, *V*, of a sample, is determined based on the frequency shift $\nu_{B}$ from an incident beam frequency in the Brillouin scattering spectrum.

(8) $$V=\frac{\lambda_{i}v_{B}}{2\;n\;\sin\frac{\theta }{2}},$$

where, *λ_i_*, *θ*, and *n* are the wavelength of an incident light, the scattering angle, and the refractive index of a sample, respectively. Using the longitudinal acoustic (LA) velocity and transverse acoustic (TA) velocity, all elastic moduli of a material can be calculated. The sound attenuation, α, is determined by.

(9) $$\alpha =\frac{\pi \Gamma }{V},$$

where Γ is the FWHM of the Brillouin peak. 

**Figure 5:**
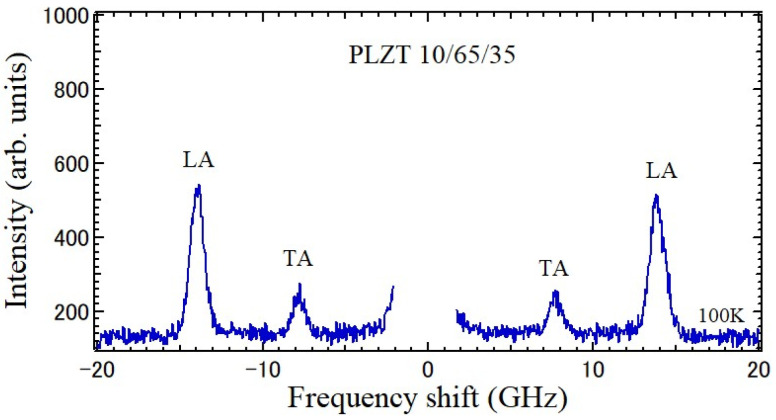
Brillouin scattering spectrum of a piezoelectric PLZT 10/65/35 ceramic at 100 K. Stokes and anti-Stokes components are observed. LA and TA denote peaks of longitudinal acoustic and transverse acoustic modes, respectively [27].

PLZT is a well-known transparent piezoelectric material, while it is very difficult to grow single crystals. Therefore, PLZT ceramics have been used for the piezoelectric application. The Brillouin scattering spectrum of piezoelectric PLZT ceramics using scanning tandem multi-pass Fabry–Perot interferometers is shown in Figure 5, in which LA and TA peaks were observed [27]. The frequency shift, $\nu_{B}=\nu_{i}-\nu_{s}\;$ > 0, is the Stokes component and the shift, $\nu_{B}=\nu_{i}-\nu_{s}\;$ < 0, is the anti-Stokes component, where $\nu_{i}\;$ and $\nu_{s}$ are the frequencies of the incident and scattered light, respectively.

For the rapid acquisition of a Brillouin scattering spectrum, the angular dispersive Fabry–Perot interferometer with a solid etalon has been developed [26].

**Figure 6:**
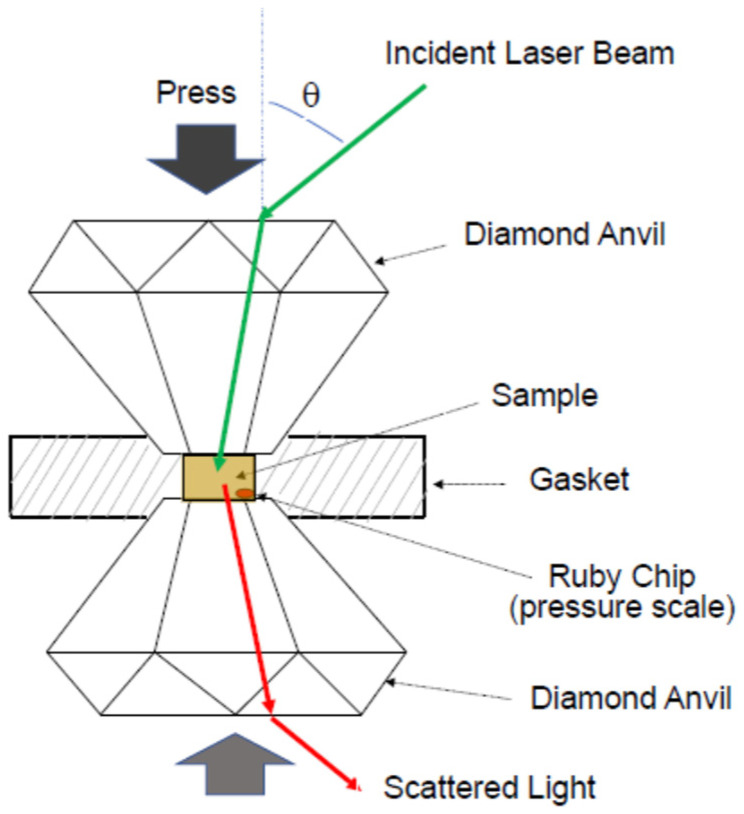
The diamond anvil cell to measure Brillouin scattering from a sample under high pressures [26]. The pressure of a sample is measured based on the pressure shift of the R1 line fluorescence of a ruby scale. Poisson’s ratio is determined based on the measurement of LA and TA velocities.

For the measurement of the pressure-dependence of Poisson’s ratio, diamond anvils are convenient to observe Brillouin scattering spectra under very high pressure, as shown in Figure 6 [26,28]. By pressing the spacing between two diamond anvils, very high pressure, of up to 100 GPa, can be applied to a sample to be studied in a gasket hole. For a solid sample, a pressure-transmitting medium is used. The pressure in a gasket hole is measured based on the pressure shift of the R_1_ line fluorescence of a ruby chip [29]. The ruby fluorescence pressure scale is the standard method to measure pressure within a sample chamber of a diamond anvil cell apparatus.

### 2.5. Atomic Force Microscopy

The atomic force microscope (AFM) is a combined system of the scanning tunneling microscope and the stylus profilometer. An image of AFM is obtained based on the measurement of the force on a sharp tip related to the proximity to the surface of a sample. In air, a lateral resolution of 30 A and a vertical resolution less than 1 A were obtained [30]. Hamazaki et al. used AFM to study the surface morphology of the ferroelastic domains BaTiO_3_. An undulation of the surface was observed around the boundary of ferroic 90° domains and was reasonably explained based on the ferroelastic strain [31]. Hurley and Turner reported the contact-resonance atomic force microscopy (AFM) methods to quantitatively measure Poisson’s ratio and the shear modulus at the same time as Young’s modulus [32]. Yamazaki et al. reported the principle of the AFM to estimate the elasticity of the cell from force curves obtained via the AFM measurement [33]. Arnold et al. reported micro-indentation methods to measure the elastic modulus of murine articular cartilage using AFM [34]. Force curves were analyzed based on fitting using the following equation with the Hertzian model.

(10) $$Y=\frac{3\left(1-\nu^{2}\right)}{4R^{1/2}\delta^{3/2}}F.$$

Here, *F*, *R*, and δ are the indentation force, probe radius, and indentation depth, respectively. They determined Young’s modulus with the assumption of *ν* = 0.5 and did not determine Poisson’s ratio independently. Further improvement is necessary for the accurate determination of Poisson’s ratio based on the use of AFM.

## 3. Poisson’s Ratio of Glasses

Using the observed values of LA velocity, *V*_L_, and TA velocity, *V*_T_, the following elastic moduli were calculated using the density, *ρ*, of a sample.

(11) $$\mathrm{Shear}\;\mathrm{modulus}:\;\;G=\rho V_{T}^{2},$$

(12) $$\mathrm{Longitudinal}\;\mathrm{modulus}:\;L=\rho V_{L}^{2},$$

(13) $$\mathrm{Bulk}\;\mathrm{modulus}:\;B=L-\frac{4}{3}G,$$

Compressibility: κ = 1/B.(14)

(15) $$\mathrm{Young}’s\;\mathrm{modulus}:\;Y=\;\frac{G\left(3L-4G\right)}{L-G},$$

(16) $$\mathrm{Poisson}’s\;\mathrm{ratio}:\;\nu =\frac{1}{2}\frac{V_{L}^{2}-{2V}_{T}^{2}}{V_{L}^{2}-V_{T}^{2}}$$

### 3.1. Composition-Dependence of Poisson’s Ratio

**Figure 7:**
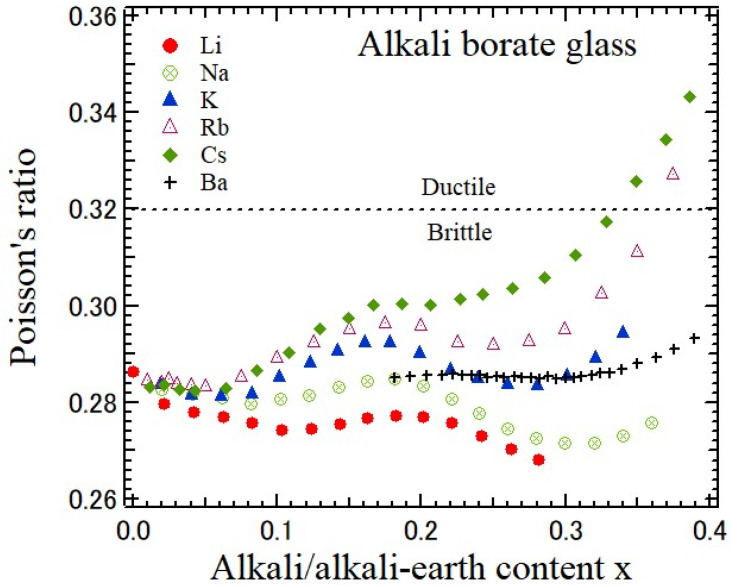
Composition-dependence of Poisson’s ratio of alkali and alkali-earth borate glasses [35,36].

In structural glasses, Poisson’s ratio is closely related to connectivity and the atomic packing density [1]. Poisson’s ratio increases as the connectivity decreases and the atomic packing density increases. In the case of oxide glasses, the number of bridging oxygen atoms, $n_{BØ}$, per glass-forming cation, such as Si, B, and P, is closely related to network crosslinking, where subscript BØ denotes bridging oxygens. For As_2_O_3_, B_2_O_3_, and P_2_O_5_ glasses, $n_{BØ}$ = 3 and *ν* ≈ 0.3, whereas for SiO_2_ and GeO_2_ glasses, $n_{BØ}$ = 4 and *ν* ≈ 0.2 [1]. Poisson’s ratio of alkali and alkali-earth borate glasses determined using the ultrasonic pulse-echo method is shown as a function of alkali and alkali-earth content in Figure 7 [35,36]. In undoped borate glass, *ν* = 0.28 is close to the case of $n_{BØ}$ = 3 and *ν* ≈ 0.3. There are two origins for the effect of alkali and alkali-earth modifications. (1) The presence of BØ_4_^−^ units increases $n_{BØ}$, and *ν* decreases. However, the BOØ_2_^−^ units decrease $n_{BØ}$, and *ν* increases, where O denotes nonbridging oxygens. (2) The BØ_4_^−^ units in pentaborate groups do not contribute to the connectivity of the surrounding structural units, and *ν* does not change. For lithium and sodium borate glasses, in the (a) first alkali content range, 0 < *x* < 0.08, *ν* decreases based on the formation of BØ_4_^−^ units. In the (b) second range, 0.08 < *x* < 0.19, pentaborate groups are formed and *ν* increases. In the (c) third range, 0.19 < *x* < 0.30, the presence of BØ_4_^−^ units increases and *ν* decreases again. In the (d) fourth range of sodium borate glass, 0.30 < *x* < 0.40, pentaborate and tri-borate groups are formed. The slight increase in BOØ_2_^−^ units causes the increase in *ν*. For potassium, rubidium, and cesium borate glasses, the formation of BOØ_2_^−^ units increases, while the formation of BØ_4_^−^ units decreases in the order from potassium to cesium borate glasses, and *ν* is larger than that of lithium and sodium borate glasses. The alkali-earth dependence of *ν* in barium borate glasses is mild, while it has a similarity with that of potassium borate glasses [36]. The variation in structural units may be smaller than that of alkali borate glasses. In cesium borate glass, Figure 7 shows the remarkable increase or Poisson’s ratio as the lithium content *x* increases and the correlation between Poisson’s ratio and fragility was observed [36]. Poisson’s ratio exceeds 0.32 for *x* > 0.34. Here, *ν* = 0.32 is the threshold of the brittle-to-ductile transition in borate glasses [37].

An overall increase in *ν*, increasing fragility m and C_g_, was suggested [3]. A remarkable increase in fragility was reported in lithium borate glasses from *m* = 32 at *x* = 0 to *m* = 60 at *x* = 0.25 based on the increase in the lithium content *x* [36]. However, Poisson’s ratio of lithium borate glass decreases as the lithium content decreases, and further discussion is necessary for the correlation between Poisson’s ratio and fragility.

### 3.2. Pressure-Dependence of Poisson’s Ratio

**Figure 8:**
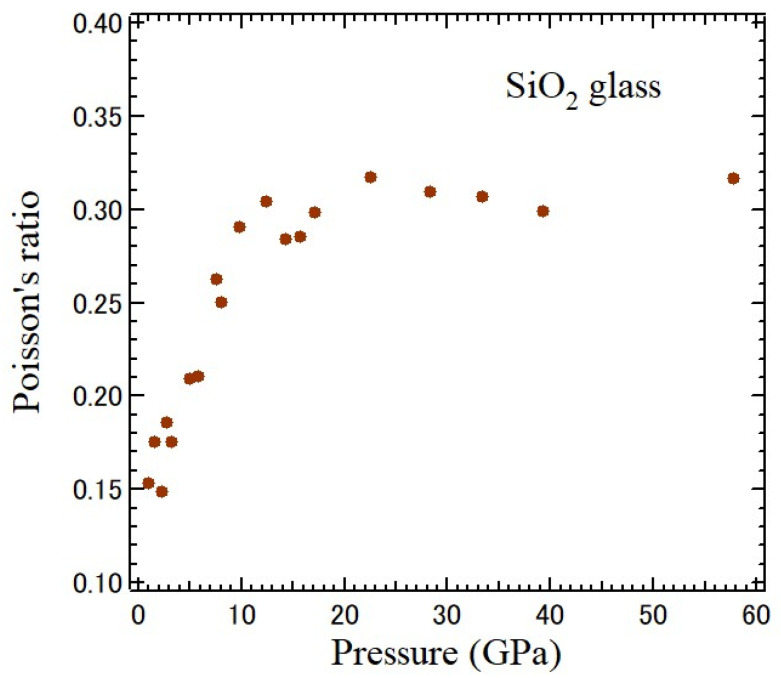
Pressure-dependence of Poisson’s ratio of an SiO_2_ glass measured based on Brillouin scattering using DAC [38].

Poisson’s ratio is very sensitive to pressure because pressure directly changes the interatomic distance. The pressure-dependence of Poisson’s ratio of a typical strong SiO_2_ glass was studied based on Brillouin scattering with a diamond anvil cell up to 57.5 GPa, as shown in Figure 8 [38]. The initial value of *ν* = 0.19 at 0.54 GPa decreases down to 0.15 at first and then increases to about 0.30–0.35. It is nearly pressure-independent above 23 GPa. At a low pressure, SiO_2_ glass is less ductile, while at a high pressure above 23 GPa, it shows typical ductility, which is like metals. These results suggest various metastable glassy states with a change in Si coordination under high pressure.

**Figure 9:**
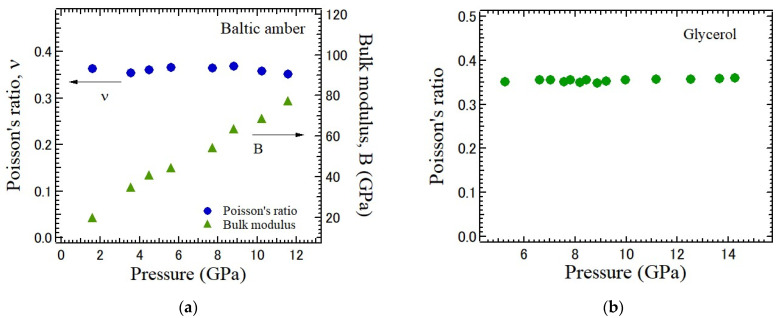
Pressure-dependence of (**a**) Poisson’s ratio ν and the bulk modulus B of Baltic amber measured based on Brillouin scattering [26], and (**b**) pressure-dependence of Poisson’s ratio of glycerol [39].

The pressure-dependence of Poisson’s ratio of typical natural fragile glass, Baltic amber, was studied [26]. Amber is a few tens of millions of years old, and it also shows ductility. The pressure-dependence of LA and TA peaks was measured based on Brillouin scattering using a diamond anvil cell. The LA frequency shift, which is proportional to LA velocity, remarkably increases as the pressure increases. The pressure-dependences of Poisson’s ratio and the bulk modulus are shown in Figure 9a. It is found that the bulk modulus monotonically increases as the pressure increases. However, the pressure-dependence of Poisson’s ratio is very small, and their values of Poisson’s ratio are about 0.35–0.37 between 1 and 12 GPa. The pressure-dependence of Poisson’s ratio of glycerol is also shown in Figure 9b [39]. Glycerol undergoes a liquid–glass transition at about 5 GPa, and above this pressure, the TA mode was observed in a glassy state using Brillouin scattering. Poisson’s ratio was determined between 5 and 14 GPa from the TA and LA sound velocities. It is found that Poisson’s ratio of glycerol in a glass state is close to that of amber, and the pressure-dependence is also very small. In general, the pressure-dependence of Poisson’s ratio of organic glasses is much smaller than that of inorganic oxide glasses.

## 4. Poisson’s Ratio of Piezoelectric Ceramics

When a crystal structure has no center of symmetry, the application of an electric field induces a strain, and the application of stress induces an electric polarization. This reversible phenomenon is called the piezoelectric effect. In free energy, piezoelectricity is given by the bilinear coupling between the strain and electric polarization. The piezoelectric effect is defined by the strain, *e_ij_*, electric flux density, *D_i_*, stress, *X_ij_*, and electric field, *E_i_*, based the following equation:

(17) $$e_{ij}=s_{ijkl}X_{kl}+d_{ijh}E_{h},\;D_{i}=\varepsilon_{ij}E_{j}+d_{ijk}X_{jk}$$

where *d_ijk_*, *s_ijkl_*, and *ε_ij_* are the piezoelectric, elastic compliance, and dielectric constants, respectively. Since the piezoelectricity is related to the third polar tensor, it disappears in a centrosymmetric crystal. Poisson’s ratio of piezoelectric materials can be measured using the piezo-resonance method described in Section 2.3 because the mechanical resonance is directly excited by a piezoelectric sample without the contact of a piezoelectric transducer.

Ferroelectric and piezoelectric materials are very important functional materials with sensing and actuating capabilities related to many applications. In this section, experimental results of Poisson’s ratio of well-known ferroelectric and piezoelectric PZT and PLZT ceramics are described.

### 4.1. Lead Zirconate Titanate Ceramics

A solid solution of *x*PbZrO_3_-(1−*x*)PbTiO_3_, PZT100*x*/100(1−*x*), is formed based on the partial replacement of Ti in ferroelectric PbTiO_3_ by Zr, where *x* is a molar ratio. PZT has a perovskite structure with the random occupancy of Ti and Zr at the B-site. Upon cooling from a high temperature, 1 wt% Nb-doped PZT75/25 (*x* = 0.75) and PZT95/5 (*x* = 0.95) ceramics undergo a structural phase transition from a paraelectric cubic to ferroelectric rhombohedral phase at *T*_C_ = 543 and 476 K, respectively [40]. For further cooling, PZT75/25 and PZT95/5 undergo a ferroelectric rhombohedral high-temperature phase, F_R_(HT), to a ferroelectric rhombohedral low-temperature phase, F_R_(LT), at about *T*_tr_ = 335 and 332 K, respectively. The F_R_(LT)–F_R_(HT) phase transition in PZT95/5 is related to two transitions, i.e., the transition from the ordered R-type to disordered R-type tilts and the one from the ordered R-type to ordered M-type tilts. However, the transition in PZT75/25 is only related to the former transition of R-type to M-type tilts.

**Figure 10:**
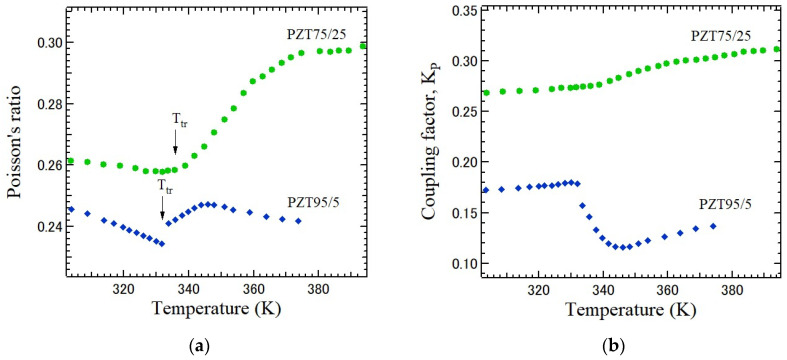
Temperature-dependences of (**a**) Poisson’s ratio and (**b**) coupling factor of piezoelectric PZT75/25 and PZT 95/5 ceramics with a perovskite structure in a ferroelectric phase [40]. *T*_tr_ is the transition temperature from F_R_(LT) to F_R_(HT).

The piezoelectric and elastic properties of PZT75/25 and PZT95/5 ceramics were studied using the piezo-resonance method [24]. Poisson’s ratio *ν* can be derived using Equation (7) from the resonance frequency, f_r_, anti-resonance frequency, f_a_, and the first overtone frequency, f_s1_. The temperature-dependences of Poisson’s ratio and electromechanical coupling factor of the radial mode K_p_ of PZT75/25 and PZT95/5 ceramics are shown in Figure 10a and Figure 10b, respectively [40]. Poisson’s ratio and the coupling factor of PZT95/5 exhibit first-order phase transition behaviors with an abrupt change at *T*_tr_. However, those of PZT75/25 exhibit a diffusive F_R_(LT)–F_R_(HT) phase transition with gradually continuous changes near *T*_tr_. These results suggest that the F_R_(LT)–F_R_(HT) phase transition of PZT75/25 is probably related to the transition between ordered R-type and disordered R-type tilts and does not include M-type tilts. Very recently, the glass-like behavior of PZT was reported in the ferroelectric rhombohedral phases and paraelectric cubic phases. This fact suggests the strong phonon damping in these phases of PZT [41]. Further studies are necessary to clarify the origin of such structural phase transitions, especially in the Zr-rich compositions.

### 4.2. Lead Lantanum Zirconate Titanate Ceramics

**Figure 11:**
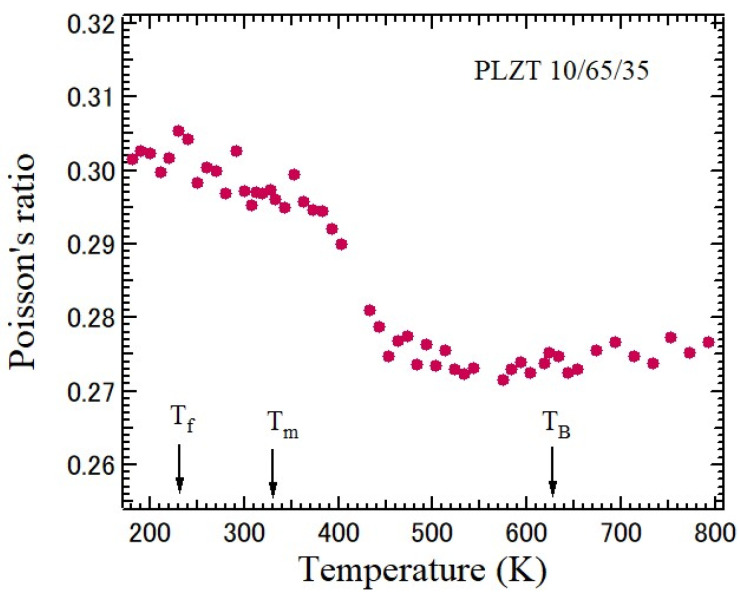
Temperature-dependence of Poisson’s ratio of piezoelectric PLZT10/65/35 ceramics with a perovskite structure [27]. It undergoes a normal-to-relaxor ferroelectric phase transition.

Lanthanum-modified lead zirconate titanate (Pb_1−*x*_La*_x_*)(Zr*_y_*Ti_1−*y*_)O_3_ (PLZT*x*/*y*/1−*y*) solid solutions with perovskite structures are transparent piezoelectric ceramics, which have various potential applications, such as electro-optical devices [42]. The compositions of the most extensively studied are *x*/65/35 with La contents from 5 to 14 at. % exhibiting a typical relaxor ferroelectric behavior. Its random fields are stronger than those of PZT. Since PLZT ceramics are transparent, elastic properties were studied based on Brillouin scattering in the large temperature range from 4 to 800 K [27]. The Brillouin scattering spectrum is shown in Figure 5. TA and LA peaks were observed, and the temperature-dependence of *V*_T_ and *V*_L_ was determined. The temperature-dependence of Poisson’s ratio of PLZT10/65/35 determined based on *V*_T_ and *V*_L_ is shown in Figure 11. 

PLZT10/65/35 undergoes a normal-to-relaxor ferroelectric phase transition upon cooling from a high temperature. The Burns temperature, *T*_B_, dielectric maximum temperature, *T*_m_, and freezing temperature, *T*_f_, are 627 K, 328 K, and 230 K, respectively. Upon cooling from a high temperature, dynamic polar nano regions (PNRs) appear at *T*_B_, which is a few hundred degrees higher than the ferroelectric Curie temperature, *T*_C_. At above *T*_B_, *ν* = 0.27, and upon cooling, it increases up to 0.295 at *T*_m_, which is close to *T*_C_. The increase *ν* can be attributed to the growth and the dynamic-to-static transition of PNRs. The diffusive change of Poisson’s ratio may be attributed to the dynamic–static transitions of PNRs. For further cooling, it becomes nearly temperature-independent, because the PNRs were frozen, and *ν* is about 0.30. For all the temperatures, *ν* is larger than 0.27.

## 5. First-Principles Calculation of Poisson’s Ratio of Crystals

The elastic, electronic, and optical properties of the newly hypothesized perovskite compound ACuO_3_ (A = Ca, Sr) were investigated under hydrostatic pressure using the first-principles method using CASTEP code [43] in the framework of density functional theory (DFT) [44]. The electronic interactions between ions and the electron are described based on the application of the exchange-correlational energy function in which the generalized gradient approximation (GGA) method developed by Perdew–Burke–Ernzerhor (PBE) is used [45]. Based on the plane-wave cut-off and k-point mesh, the convergence of the present calculations was examined carefully. The ground state structure was calculated using the Brodyden–Fletcher–Goldfarb–Shanno (BFGS) method [46]. A smooth and computation-friendly pseudopotential was produced based on ultrasoft Vanderbilt-type pseudopotentials, and it saves computation time significantly without affecting the accuracy appreciably. The independent elastic constants and elastic moduli were calculated using the stress–strain method using the CASTEP code [47]. 

For a cubic system, the elastic moduli, *Y*, *B*, *G*, and Poisson’s ratio, *ν*, were determined using the calculated elastic stiffness constants, *C_ij_*. By averaging the upper and lower bounds of Voigt’s and Reuss’s techniques, *B* and *G* have been calculated based on the Voigt–Reuss–Hill approximation [48] using the following equations:

(18) $$B_{V}=B_{R}=\frac{1}{2}\left(C_{11}+2C_{12}\right).$$

(19) $$G_{V}=\frac{1}{5}\left(C_{11}-C_{12}+3C_{44}\right).$$

(20) $$G_{R}=\frac{5\left(C_{11}-C_{12}\right)C_{44}}{C_{44}+3\left(C_{11}-C_{12}\right)}.$$

(21) $$B=\frac{1}{2}\left(B_{V}+B_{R}\right).$$

(22) $$G=\frac{1}{2}\left(G_{V}+G_{R}\right).$$

where the subscript *R* denotes the Reuss approximation, the subscript *V* denotes the Voigt approximation, and *B* and *G* have been calculated following the Voigt–Reuss–Hill approximation.

In addition, *Y* and *ν* are calculated based on the following equations [47].

(23) $$Y=\frac{9BG}{3B+G}.$$

(24) $$v=\frac{3B-2G}{2\left(3B+G\right)}.$$

**Figure 12:**
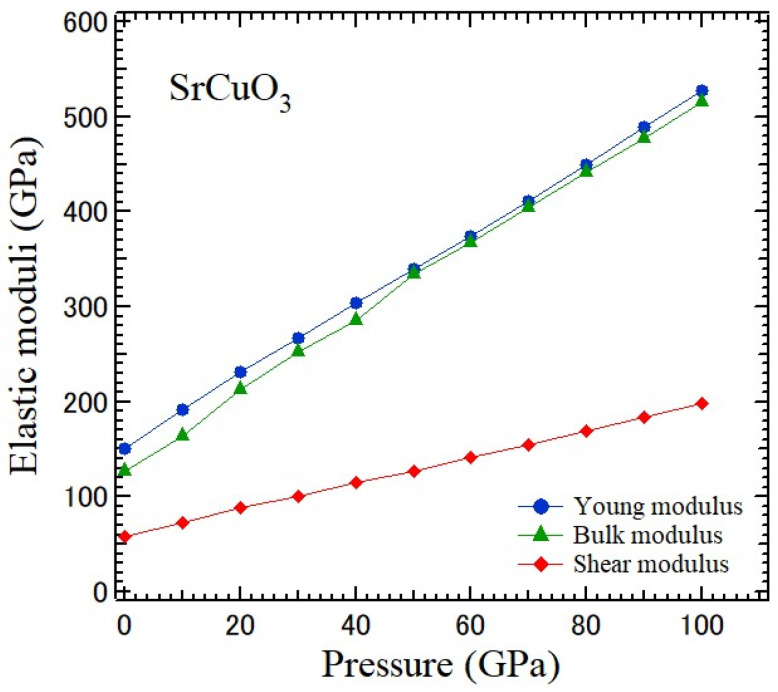
Pressure-dependences of Young, bulk, and shear moduli of an SrCuO_3_ crystal.

Poisson’s ratio is the essential mechanical indicator. Since brittle materials can bear much stress, they are tough. However, they cannot stretch much and may break down suddenly. The stress–strain relationship of ductile materials is linear, and their elastic regions are larger. The critical value *ν* to determine the ductile/brittle nature of solids is 0.26 [49]. Figure 12 shows the pressure-dependences of the Young, bulk, and shear moduli of an SrCuO_3_ crystal.

**Figure 13:**
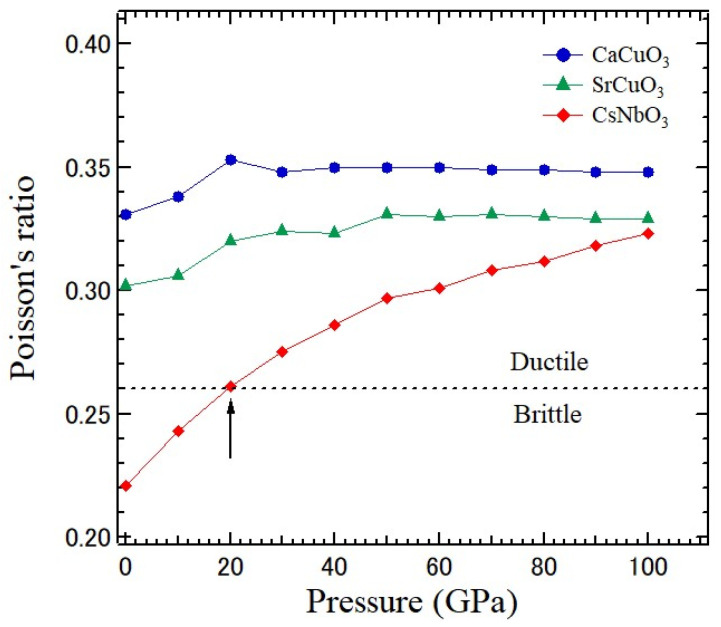
Pressure-dependence of Poisson’s ratio of CaCuO_3_, SrCuO_3_, and CsNbO_3_ crystals determined based on the first-principles calculation [47,50].

Figure 13 shows the pressure dependence of Poisson’s ratio, *ν*, of CaCuO_3_, SrCuO_3_, and CsNbO_3_ crystals. For CaCuO_3_ and SrCuO_3_ crystals, values *ν* at different pressures are larger than 0.26. This fact indicates that the compounds CaCuO_3_ and SrCuO_3_ are ductile. The degree of ductility of two crystals shows nearly a monotonic behavior with applied pressure. The sort of interatomic forces involved in a crystal is also determined by the value of *ν*. When the value *ν* is between 0.25 and 0.50, the central force interaction is dominant and non-central. Since the values of *ν* in CaCuO_3_ and SrCuO_3_ are between 0.25 and 0.50 for all pressures, the interatomic forces can be central in both materials. In a comparison of the indicator, it is confirmed that CaCuO_3_ is more ductile than SrCuO_3_.

The cubic phase of a CsNbO_3_ crystal with a perovskite structure was also hypothesized to investigate the elastic, electronic, photocatalytic, and optical properties related to applications in industries using the first-principles method [50]. Frantsevich et al. reported, at first, the separation of ductility from brittleness of materials on the basis of Poisson’s ratio [51]. Frantsevich’s rule suggested that *ν* = 0.26 is the divided line between the brittle and ductile materials. If *ν* > 0.26, then the material is ductile and plastic deformation occurs. If *ν* < 0.26, the material is brittle and no plastic deformation occurs. The values of *ν* above 20 GPa indicated a ductile nature. Meanwhile, the values below 20 GPa show a brittle nature, as shown in Figure 13.

## 6. Conclusions

Poisson’s ratio is a very important elastic modulus used to discuss various properties of glasses, ceramics, and crystals. It is related to the connectivity, mean coordination number, valence electron density, and fragility of glass-forming materials. At first, the five kinds of experimental methods to determine Poisson’s ratio are introduced, namely the ultrasonic pulse-echo method, resonant ultrasonic spectroscopy, piezoelectric resonance method, Brillouin scattering spectroscopy, and atomic force microscopy. For the evaluation of elastic constants of hypothesized compounds, the first-principles calculation is described. Poisson’s ratio is sensitive to the composition, temperature, and pressure. The experimental results based on the dependences of Poisson’s ratio on the temperature, pressure, and composition are reviewed for various glasses, ceramics, and crystals. The mechanism of these variations is discussed from physical and chemical points of view. In oxide glasses, the number of bridging oxygen atoms per glass-forming cation is directly related to network crosslinking through Poisson’s ratio. The correlation between Poisson’s ratio and fragility is discussed. Piezoelectric ceramics show the anomaly in the vicinity of a structural phase-transition temperature. Based on the first-principles calculation, ductile–brittle transition is discussed.

## Data Availability

The data that support the findings of this study are available from the corresponding author upon reasonable request.
